# Efficient CRISPR/Cas9 genome editing with Citrus embryogenic cell cultures

**DOI:** 10.1186/s12896-020-00652-9

**Published:** 2020-11-10

**Authors:** Manjul Dutt, Zhonglin Mou, Xudong Zhang, Sameena E. Tanwir, Jude W. Grosser

**Affiliations:** 1 Citrus Research and Education Center, University of Florida, Lake Alfred, Florida, USA; 2 Department of Microbiology and Cell Science, University of Florida, Gainesville, Florida, USA

**Keywords:** *Agrobacterium tumefaciens*, CRISPR/Cas9, Citrus cell suspensions, Genetic transformation

## Abstract

**Background:**

Development of precise genome editing strategies is a prerequisite for producing edited plants that can aid in the study of gene function and help understand the genetic traits in a cultivar. Citrus embryogenic cell cultures can be used to rapidly produce a large population of genome edited transformed citrus lines. The ability to introduce specific mutations in the genome of these cells using two constructs (pC-PDS1 and pC-PDS2) was evaluated in this study.

**Results:**

*Citrus sinensis* ‘EV2’ embryogenic cell cultures are amenable to *Agrobacterium*-mediated CRISPR/Cas9-based genome editing. Guide RNAs (gRNAs) targeting two locations in the phytoene desaturase (*PDS*) gene were either driven by the *Arabidopsis* U6–26 promoter (pC-PDS1) or assembled as a Csy4 array under the control of the CmYLCV promoter (pC-PDS2). All transgenic embryos were completely albino and no variegated phenotype was observed. We evaluated 12 lines from each construct in this study and the majority contain either insertion (1–2 bp), substitution (1 bp), or deletion (1–3 bp) mutations that occurred close to the protospacer adjacent motif.

**Conclusions:**

Both the pC-PDS1 and pC-PDS2 could successfully edit the citrus embryogenic cell cultures. However, the editing efficiency was dependent on the gRNA, confirming that the selection of a proper gRNA is essential for successful genome editing using the CRISPR/Cas9 technique. Also, utilization of embryogenic cell cultures offers another option for successful genome editing in citrus.

## Background

Citrus is a perennial fruit crop susceptible to a plethora of abiotic and biotic stresses. Considerable efforts have been made to develop superior citrus cultivars that can better withstand abiotic and biotic stresses and at the same time produce optimum yields using conventional breeding and genetic modification strategies. Owing to the high heterozygosity and long juvenility of many citrus cultivars, conventional breeding approaches can often be tedious and time-consuming. Genetic transformation, on the other hand, can provide rapid solutions and is becoming increasingly popular [1–5].

CRISPR/Cas9 has emerged as the most extensively used genome-editing system in the recent years [6, 7]. This tool is based on the bacterial clustered regularly interspaced short palindromic repeats (CRISPR)/CRISPR-associated protein 9 (Cas9) system [6, 7]. In addition to the pioneering studies in *Arabidopsis* [8] and *Nicotiana* [9–11], the CRISPR/Cas9 system has been successfully used to target specific genomic sequences of interest for the development of genome edited citrus [12–16].

Citrus can be transformed using different explant sources – both juvenile and mature tissue derived. Among the juvenile explants, epicotyl tissues [17], embryogenic cell cultures [18] and protoplasts [19] are commonly used. Mature stem pieces have also been used to produce transgenic citrus [20] and this process bypasses the juvenile stage to produce transformed trees that can flower and fruit within 12–18 months after regeneration. In cases where juvenile epicotyl or mature stem tissues are the target explants for *Agrobacterium* mediated transformation, a high frequency of chimeric shoots are commonly produced [21]. Additionally, a large population of non-transformed escapes are also observed while using these source explants [22]. Genetic transformation and regeneration of transgenic plants through the process of somatic embryogenesis (utilizing embryogenic cell cultures or protoplasts) results in enhanced genetic transformation efficiency and is especially suitable for transforming seedless citrus cultivars [18, 23].

To study the efficacy of the CRISPR/Cas9 technique in plants, the phytoene desaturase (*PDS*) gene is commonly targeted. The disruption of this gene impairs chlorophyll and carotenoid production resulting in an albino phenotype that can be observed visually [24] to estimate the efficacy of the genome modification system. Mutations induced in the *PDS* gene can result in a clear albino phenotype at a high frequency in citrus [15]. Mutations in the *PDS* gene introduced by CRISPR/Cas9 have also been shown to confer an albino phenotype in other fruit crops such as apple [25, 26], grapes [27], kiwifruit [28], pear [25], and kumquat [29].

To fully expand the potential of the CRISPR/Cas9 system in citrus, we evaluated a CRISPR construct that encoded gRNAs driven by the *Arabidopsis* U6–26 pol III promoter and compared it to a construct that harnessed the Csy4 bacterial endoribonuclease's RNA processing ability. We subsequently transformed citrus suspension cells of a recently released low seeded sweet orange cultivar with the two constructs, each targeting two locations in the *CsPDS* gene. Our results are expected to lay the groundwork for the development of edited plants in most citrus cultivars, especially seedless cultivars that have not been edited using other explant sources.

## Methods

### Construction of CRISPR/Cas9 vectors

**Fig. 1:**
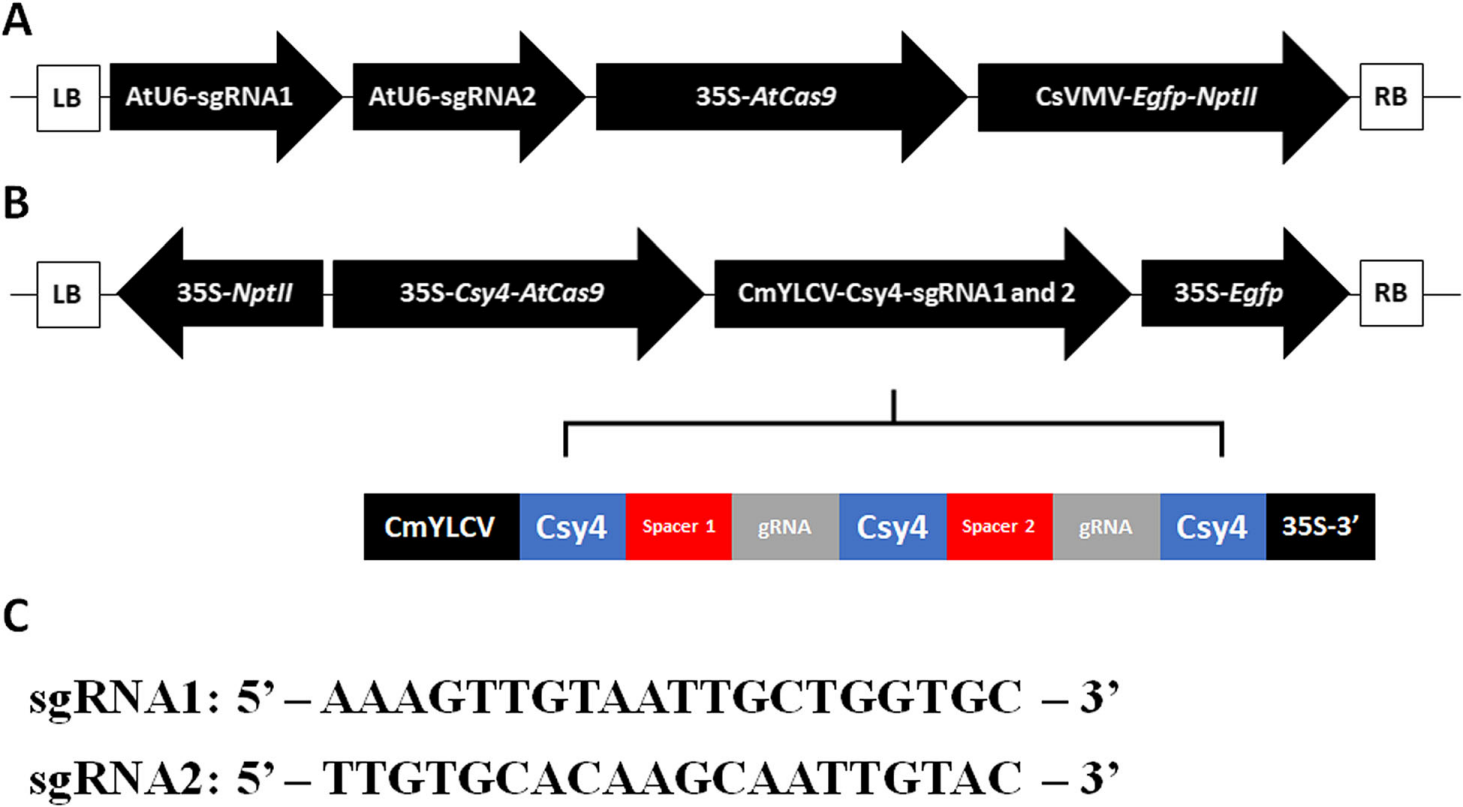
Embryogenic cell culture mediated CRISPR/Cas9 genome editing in citrus. **a** Schematic representation of the pC-PDS1 construct; **b** Schematic representation of the pC-PDS2 construct; **c** The 20 bp sequences of the sgRNAs targeted in this study

Two constructs were generated in this study, each containing the two gRNA sequences, gRNA1: 5′-AAAGTTGTAATTGCTGGTGC-3′ [15] and gRNA2: 5′-TTGTGCACAAGCAATTGTAC-3′ [12]. The first was based on the pCAMBIA2300-GFP vector [18]. This vector was modified to contain a 35S promoter-driven *AtCas9* gene. The *Arabidopsis* U6–26 promoter was utilized to drive the expression of the gRNAs in this vector. The resulting plant transformation vector was called pC-PDS1 (Fig. 1a). The NEBuilder® HiFi DNA Assembly Cloning Kit (NEB, Ipswich, MA, USA) was used as per the manufacturer’s protocols to produce pC-PDS1.

The second construct was based on the empty backbone vector pTrans_223, which is a plant expression vector based on the pCAMBIA backbone [30]. The gRNAs were initially cloned into the modular vector pMOD_B2103 which contains the CmYLCV promoter [31] with a SapI ccdb cassette for cloning the multiple gRNA spacers with Csy4 spacers and followed by the 35S terminator, resulting in pMOD_B2103_Csy4/gRNA. This vector and the modular vectors pMOD_A0501 (35S promoter driving the Csy4-P2A-AtCas9 gene with an AtHSP terminator) and pMOD_C3001 (35S promoter driving the GFP gene with a pea rbcsE9 terminator) were subsequently assembled into pTrans_223 using the Golden Gate assembly method [30, 32]. The resulting plant transformation vector was called pC-PDS2 (Fig. 1b). All modular vectors for building pC-PDS2 were obtained from Addgene (https://www.addgene.org). The primers used in this study were synthesized by Integrated DNA Technologies, Inc. (Coralville, IA, USA). All constructs were verified using the Sanger sequencing method and introduced into *Agrobacterium tumefaciens* EHA105 by the freeze-thaw method [33].

### *Agrobacterium* mediated transformation of embryogenic cell cultures

Embryogenic callus was initiated from the unfertilized ovules of *Citrus sinensis* (L.) Osbeck cv. ‘EV2’ [34]. Actively dividing one-year-old embryogenic callus, sub-cultured at 1-month intervals on DOG medium [35], was used to initiate suspension cells that were used in this study. The suspension cells were maintained as described by Kaur et al. [36]. *A. tumefaciens* cells initiated overnight in YEP medium were spun down and resuspended in liquid DOG medium to a final OD of 0.3. Approximately five grams of the suspension cells were then incubated in this solution for 20 min before being dried on a stack of sterile filter paper disks, essentially as described by Dutt and Grosser [18]. After a 5-day co-cultivation in solid EME medium supplemented with maltose (EME-M) and 100 μM acetosyringone, the cells were washed to remove residual *Agrobacterium* and plated on EME-M supplemented with appropriate antibiotics. The cells were maintained in the dark for 2 weeks before transfer to a standard 16-h light/8-h dark cycle. Developing embryos that were white in color were observed for EGFP-specific fluorescence. The transformation efficiency percentage was calculated as the total number of EGFP-positive embryos per total number of embryos produced (EGFP-negative + positive) **×** 100.

### Detection of CRISPR/Cas9 induced *PDS* mutations

For molecular analysis, genomic DNA from EGFP positive and control citrus embryos were isolated using the DNeasy Plant Pro Kit (Qiagen, Germantown, MD). DNA was normalized to 25 ng.ml^− 1^ using a NanoDrop Spectrophotometer (Thermo Fisher Scientific Inc.). To confirm the presence of the Cas9 and EGFP transgene in the putative edited embryos, duplex PCR was carried out in a thermal cycler (C1000 Touch; Bio-Rad Laboratories, Hercules, CA) using GoTaq Green Master PCR Mix (Promega Corp, Madison WI) and primers that amplified the Cas9 (Cas-F 5′-CAGGCTCTCTGATTACGATGTT-3′ and Cas-R 5′-GCGAAATCCCTTCCCTTATC-3′; 750 bp) and EGFP (EG-F 5′-GGGTGAAGGTGATGCAACATA-3′ and EG-R 5′-GCAGATTGTGTGGACAGGTAAT-3′; 520 bp). For mutation analysis, primer pairs were designed to amplify a DNA fragment surrounding each target. A 430-bp sequence around the gRNA1 was amplified using the primers gRNA1F: 5′-TACAGGTGGTTTGTGTGGAC-3′ and gRNA1R: 5′-TCCACAATGCCATACACACC-3′. Similarly, a 397-bp sequence around the gRNA2 was amplified using the primers gRNA2F: 5′-TACAAAGGTCTCCTGTAGAAG-3′ and gRNA2F: 5′-AGCAGCACATAGTCCTGAAC-3′. OneTaq® Hot Start 2X Master Mix with standard buffer (New England Biolabs, Ipswich, MA, USA) was used for PCR and the products were either sequenced directly by the Sanger method or cloned into the pCR™4-TOPO® TA Vector (TOPO™ TA Cloning™ Kit for Sequencing, Thermo Fisher Scientific Inc.). The sequencing results were compared with the sequence of the citrus *PDS* gene by alignment using the AlignX program of the Vector NTI Advance™ software (Thermo Fisher Scientific Inc.).

## Results

**Fig. 2:**
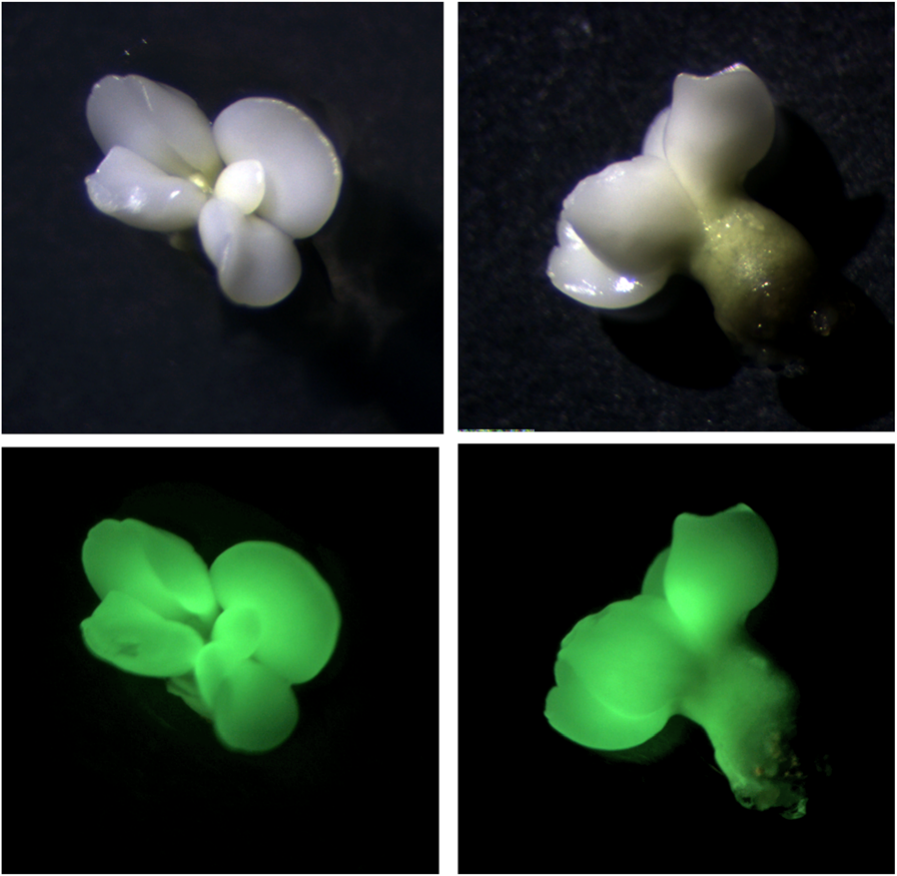
Genome-edited cotyledonary stage citrus somatic embryos with mutations in the *PDS* gene and an albino phenotype, and the same embryos below exhibiting EGFP expression under an epi-fluorescence stereomicroscope

In this study, we transformed the embryogenic cells of the recently released sweet orange ‘EV2’ cultivar with two CRISPR/Cas9 constructs (pC-PDS1 and pC-PDS2) targeting the *C. sinensis PDS* (*CsPDS*) gene (Genbank accession no. AJPS01008466.1 (10,783..29519)) and produced a population of genome edited embryos. *CsPDS* was selected due to the ability to generate visible albino phenotype in the *PDS* mutants. The two distinct gRNAs used in this study were based on earlier published reports. The first was based on the work published by Zhang et al. [15] and is located in the second exon of the *CsPDS* gene, 258 bp from the start codon. The second was based on the study by Jia and Wang [12], and is located 18,349 bp from the start codon (Fig. 1c). A large population of somatic embryos (SE) were produced from all experiments. Transgenic SE, expressing EGFP and with visible albino mutant phenotypes were regenerated in all experiments within 2–3 months following transformation (Fig. 2).

**Fig. 3:**
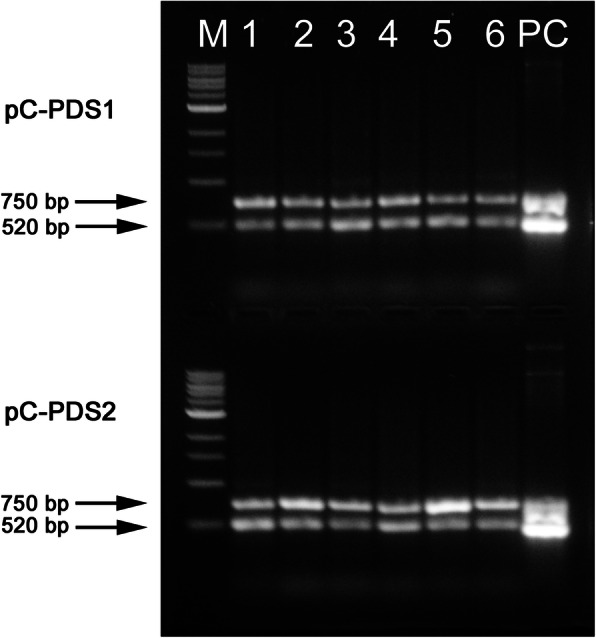
Amplification products obtained from duplex PCR of transgenic ‘EV2’ genomic DNA with gene-specific oligonucleotide primers. A 750 bp fragment of the *Cas9* gene was amplified along with a 520 bp fragment of the *egfp* gene. M, 1 kb marker; 1–6 are six individual transgenic lines containing the pC-PDS1 cassette (upper panel) and the pC-PDS2 cassette (lower panel). PC is positive plasmid DNA control

**Fig. 4:**
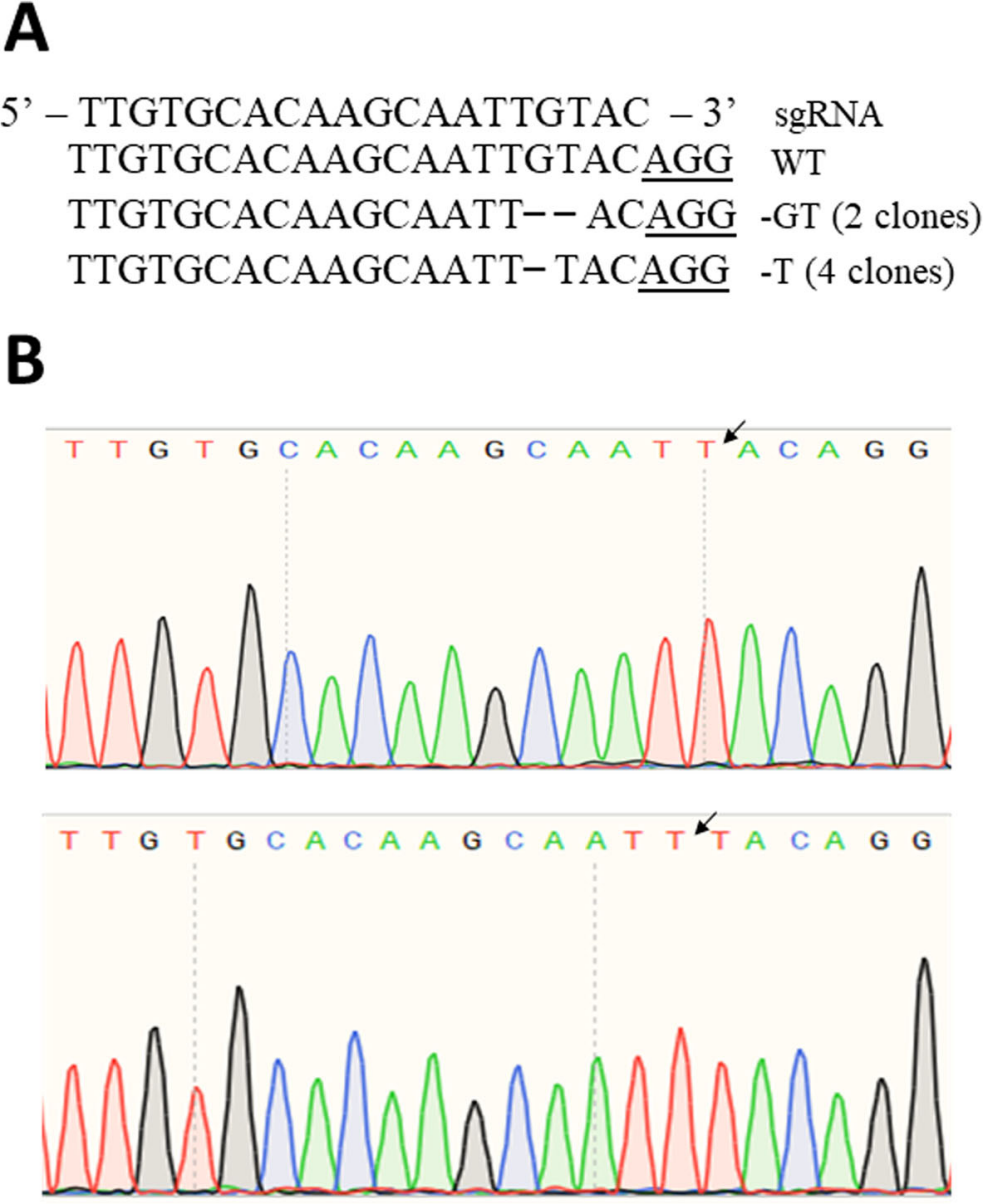
**a** Representative sequence alignment of CRISPR/Cas9-induced deletion mutations. The PAM sequence is underlined; **b** Sequencing chromatograms. Arrows indicate the site of mutation

Following transformation and successful regeneration, none of the albino SE survived for more than two successive in vitro propagation cycles. The non-transgenic embryos always remained fully green at this stage. We did not observe any chimeric SEs in any of our experiments. A total of 15 EGFP+ SEs were produced from the pC-PDS1 construct while transformation with the pC-PDS2 construct resulted in 19 EGFP+ SEs (Table 1). We isolated gDNA from 12 randonly selected transformed lines from each of the constructs for further analysis. PCR analysis of the genomic DNA determined that all the lines tested positive for the presence of both the Cas9 and the EGFP genes (6 lines from each construct were shown in Fig. 3). Sanger sequencing results of a 430 bp PCR amplified sequence around the gRNA1 and a 397 bp sequence around the gRNA2 revealed that all albino embryos carried at least one mutation in the *CsPDS* gene (Table 1). We commonly observed single base changes such as substitutions and deletions in the *CsPDS* gene and the frequency of single base changes was always greater than deletions (Fig. 4). All clones with the same mutation were classified as bi-allelic and homozygous, while clones that had different pattern of mutations, including the wild type sequence were putatively heterozygous. Our phenotypic data indicated no chimeric embryo production, which could have been easily visually observed from the sectorial chlorophyll production. Additionally, all the sequenced clones that contained the mutation were observed to be bi-allelic and homozygous.

**Table 1:** *Agrobacterium* mediated genetic transformation of embryogenic cell cultures of *Citrus sinensis* ‘EV2’

Construct	Total Somatic Embryos ± SE	EGFP+ Somatic Embryos ± SE	Transformation Efficiency (%)	Lines tested	gRNA1 mutation	gRNA2 mutation
pC-PDS1	57	15	26.3	12	12	10
pC-PDS2	52	19	36.5	12	12	11

We also observed differences in the editing efficiency between the two guide RNA sites as previously reported by Zhang et al. [15]. There was a 100% mutation rate in the gRNA1 (258 bp from the start codon) when either of the constructs was used (Table 1). However, when sequences around the gRNA2 (18,349 bp from the start codon) were evaluated, 2 lines produced with the pC-PDS1 and 1 line produced with the pC-PDS2 resembled the wild type DNA.

## Discussion

Citrus can be transformed using several explant resources and the embryogenic cell suspension system is one of the most efficient methods of juvenile tissue transformation. This is due to higher transformation efficiency, with the potential for rapid production of a large population of transgenic lines [23]. Embryogenic callus cells can be initiated from most polyembryonic citrus cultivars, and is relatively easy to establish and maintain from sweet oranges, while lemons and some mandarins produce embryogenic cells with some difficulty. The subsequent cell suspension produced from the in vitro derived cells offer the ability to produce transgenic events throughout the year, since these totipotent cells can be maintained in vitro and utilized as needed [18] and are not dependent on seasonal seed availability like the epicotyl mediated transformation process [3]. This system has not yet been explored as a tool in the CRISPR/Cas9 modification of the citrus genome and our report provides the first insight using this technique.

Albino mutant lines have been obtained in previous studies on citrus, although other explant types were used in those studies [12, 15, 29]. Similar to our observations, Zhang et al. [15] also reported that most of the mutations obtained in their study were identified as indels that resulted in a frameshift mutation. A 1 bp insertion was primarily recorded by Zhu et al. [29] in *Fortunella hindsii PDS* edited plants while both deletions and nucleotide substitutions were reported by Jia and Wang [12] following the agroinfiltration-mediated transient expression in citrus leaf tissues. Somatic embryos haven been observed to be of single cell origin in both monocots [37] and dicots [38] and our bi-allelic and homozygous mutations would suggest the single cell origin of these citrus somatic embryos. Proper choice of the gRNA is essential for efficient genome editing [39] and promising targets can be selected by in vitro DNA cleavage assay to help in improved CRISPR/Cas9 based mutagenesis [40]. Our results confirmed that successful genome editing using the CRISPR/Cas9 technique in citrus is dependent on the selection of the guide RNA sequence [41].

An advantage of using the Csy4 processing enzyme lies in the ability to produce compact transformation constructs that can simultaneously express several gRNAs [30]. The Csy4 protein is a type III CRISPR/Cas-associated protein from *Pseudomonas aeruginosa* [42] that has been extensively used in both eukaryote and prokaryote genome editing [43]. We wanted to understand the utility of this system for genome editing of citrus and our study confirms the functionality of this enzyme in citrus, when fused in frame to the Cas9 sequence to create a Csy4-Cas9 fusion protein based on that described by Čermák et al. [30]. Our results confirmed that both the U6 promoter derived system (in pC-PDS1) and the Csy4 derived system (in pC-PDS2) were comparably efficient in producing targeted mutations in the citrus genome. However, in the lines that we evaluated, we observed 2–3 bp deletions in the transgenic embryos modified using pC-PDS2, which is in contrast with either single base deletions or substitutions as observed with pC-PDS1.

## Conclusions

The findings of this study provide insight that the identification of a proper guide RNA sequence is essential in producing efficiently edited citrus plants. Both constructs utilized in our study were efficient in inducing mutations in the *CsPDS* gene. The selection of the CRISPR/Cas9 based genome editing system will depend on the final desired outcome since it is easier to target multiple genes with the Csy4 derived system. Utilization of an efficient construct coupled with an efficient transformation system can result in the production of a large population of transgenic lines that can then be screened to obtain a desired mutant line. Our embryogenic cell culture mediated transformation system is superior to the epicotyl explant mediated system, primarily because of the larger population of transformed plants that can be produced at any given point in time. Additionally, seedless citrus cultivars and many epicotyl transformation recalcitrant cultivars can be easily transformed with this system [18, 23].

## Supplementary Information

**Additional file 1: Figure S1.** Uncropped original PCR gel image of Fig. 3. The cropped area is indicated by a red dashed rectangle.

## Data Availability

The datasets used and/or analyzed during the current study are available from the corresponding author on reasonable request.

## Abbreviations

CRISPR: Clustered Regularly Interspaced Short Palindromic Repeats
EGFP: Enhanced Green Fluorescent Protein
gRNA: guide RNA
PCR: Polymerase chain reaction
PDS: Phytoene desaturase
pol III: RNA polymerase III
RNA: Ribonucleic Acid
