# Supplementary material for: Efficient CRISPR/Cas9 genome editing with Citrus embryogenic cell cultures
Source: BMC Biotechnol. 2020 Nov 10;20:58. doi: 10.1186/s12896-020-00652-9 (PMC7654154; doi:10.1186/s12896-020-00652-9)
Supplement: Supplementary file 1 — Additional file 1: Figure S1. Uncropped original PCR gel image of Fig. 3. The cropped area is indicated by a red dashed rectangle. [file 12896_2020_652_MOESM1_ESM.pdf]

Supplementary Information

Efficient CRISPR/Cas9 Genome Editing with Citrus Embryogenic Cell Cultures

Manjul Dutt[1*], Zhonglin Mou[2], Xudong Zhang[2], Sameena E. Tanwir[1], and Jude W. Grosser[1]

[1] Citrus Research and Education Center, University of Florida, Lake Alfred, Florida, United States of America.

[2] Department of Microbiology and Cell Science, University of Florida, Gainesville, Florida, United States of America.

*Corresponding author

E-mail: manjul@ufl.edu

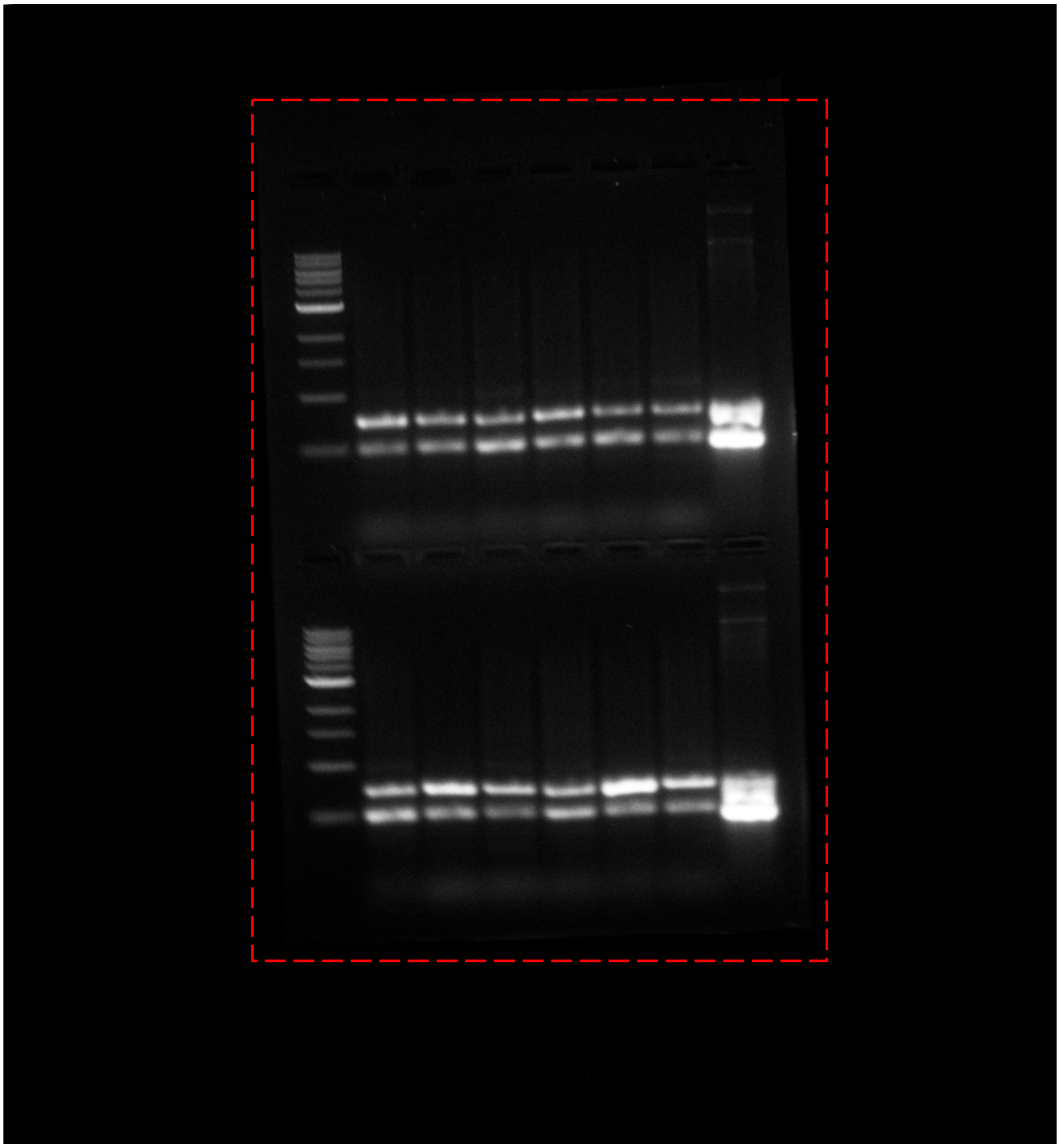

Supplementary Fig S1: Uncropped original PCR gel image of Figure 3. The cropped area is indicated by a red dashed rectangle.
